# Thyroxine increases *Serca2* and *Ryr2* gene expression in heart failure rats with euthyroid sick syndrome

**DOI:** 10.1590/2359-3997000000208

**Published:** 2016-09-26

**Authors:** Fábio V. G. Campanha, Denise Perone, Dijon H. S. de Campos, Renata de A. M. Luvizotto, Maria T. De Síbio, Miriane de Oliveira, Regiane M. C. Olimpio, Fernanda C. F. Moretto, Carlos R. Padovani, Gláucia M. F. S. Mazeto, Antonio C. Cicogna, Célia R. Nogueira

**Affiliations:** 1 Unidade de Pesquisa Experimental Faculdade de Medicina de Botucatu Universidade Estadual Paulista Botucatu SP Brasil Departamento de Clínica Médica, Unidade de Pesquisa Experimental (Unipex), Faculdade de Medicina de Botucatu, Universidade Estadual Paulista (Unesp), Botucatu, SP, Brasil; 2 Instituto de Ciências da Saúde Universidade Federal de Mato Grosso Sinop MT Brasil Instituto de Ciências da Saúde, Universidade Federal de Mato Grosso (UFMT), Sinop, MT, Brasil; 3 Departamento de Bioestatística Instituto de Biociências Universidade Estadual Paulista Botucatu SP Brasil Departamento de Bioestatística, Instituto de Biociências, Universidade Estadual Paulista (Unesp), Botucatu, SP, Brasil

**Keywords:** Reverse triiodothyronine, heart failure, therapeutic use, calcium channels, triiodothyronine

## Abstract

**Objective:**

The current study was aimed at analyzing sarcoplasmic reticulum Ca^2+^ ATPase (Serca2) and ryanodine receptor type 2 (*Ryr2*) gene expression in rats subjected to surgery that induced HF and were subsequently treated with T4 using physiological doses.

**Materials and methods:**

HF was induced in 18 male Wistar rats by clipping the ascending thoracic aorta to generate aortic stenosis (HFS group), while the control group (9-sham) underwent thoracotomy. After 21 weeks, the HFS group was subdivided into two subgroups. One group (9 Wistar rats) with HF received 1.0 µg of T4/100 g of body weight for five consecutive days (HFS/T4); the other group (9 Wistar rats) received isotonic saline solution (HFS/S). The animals were sacrificed after this treatment and examined for signs of HF. Samples from the left ventricles of these animals were analyzed by RT-qPCR for the expression of Serca2 and *Ryr2* genes.

**Results:**

Rats with HF developed euthyroid sick syndrome (ESS) and treatment with T4 restored the T3 values to the Sham level and increased Serca2 and *Ryr2* gene expression, thereby demonstrating a possible benefit of T4 treatment for heart function in ESS associated with HF.

**Conclusion:**

The T4 treatment can potentially normalize the levels of T3 as well elevated Serca2 and *Ryr2* gene expression in the myocardium in heart failure rats with euthyroid sick syndrome.

## INTRODUCTION

Euthyroid sick syndrome (ESS) is defined as a drop in the levels of triiodothyronine (T3) and increase in the levels of reverse T3 (rT3), with or without decrease in thyroxine (T4) or thyroid-stimulating hormone (TSH) (1,2) in critically infirm patients (3,4). Both hyperfunction and hypofunction of thyroid have multiple effects on the cardiovascular system; therefore, normal endocrine function is essential for cardiovascular health (5).

ESS occurs in various clinical conditions, such as malnutrition, liver or kidney failure, systemic disease, human immunodeficiency virus (HIV) infection, trauma and post-operative infection (6). Its appearance in patients with heart failure is an indicator of the severity of the disease (7). In addition, it is unclear whether the appearance of ESS is only a prognostic marker of HF or that ESS contributes to the worsening of HF (8). However, it is known that normal thyroid hormone levels are restored when the HF is resolved.

A drop in T3 levels is the most common abnormality in decompensated HF. T3 levels reduce rapidly within the first 24 hours of escalation of the disease, while rT3 levels increase and TSH and total and free T4 levels remain normal (2).

The activity of various channels and pumps located in the sarcolemma and in the sarcoplasmic reticulum (SR) regulates the transit of intracellular Ca^2^, modulating the contraction and relaxation of the myocardium. It is also known that a reduction in T3 promotes a reduction in the expression of calcium transport proteins; sarcoplasmic Ca^2^ATPase (*Serca2*) and ryanodine receptor type 2 (*Ryr2*) (9,10).

It is not clear whether treatment with thyroid hormone in patients with HF and ESS results in favorable hemodynamic changes and improves heart function (11). While some experimental work has shown favorable results after treatment (principally with T4 analogues) (10-12), other studies have shown either no difference in relation to the placebo, or an increase in the number of collateral effects while providing no benefit in relation to the placebo (13,14).

Hence, the present study assessed the effect produced by treatment of HF-induced ESS with physiological doses of T4 on *Serca2* and *Ryr2* mRNA expression in rats. We found that HF-induced ESS resulted in decreased T3 levels and a decrease in *Serca2* and *Ryr2* gene expression. T4 treatment after HF increased *Serca2* and *Ryr2* gene expression.

## MATERIALS AND METHODS

### Animal models and experimental protocol

All procedures outlined in this study were approved by the Ethics Committee of Botucatu Medical School (Unesp, SP, Brazil) and performed in accordance with the Guide for the Care and Use of Laboratory Animals published by the National Research Council in the 1996 (15).

A total of 50 male Wistar rats (aged 60 days and weighing approx. 392 g) were used for this study. The animals were initially divided into two groups: sham (10 rats submitted to simulated surgery, thoracotomy without the insertion of a clip) and HFS group (40 rats submitted to thoracotomy with the insertion of a clip). They were anesthetized posteriorly and subjected to trichotomy of the anterior median region of the thorax. After that, the animals were placed in the supine position and manual ventilation was provided by positive pressure with oxygen at 100%. A median sternotomy was carried out and a clip was placed in the case of the HFS group; the thoracic cavity was closed with 5.0 mononylon thread.

**Figure 1:**
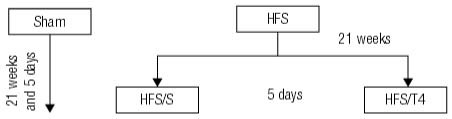
Experimental protocol.

After surgery, the animals were assessed daily in order to detect signs of HF such as tachypnea, cyanosis, hair standing on end, edema, ascites, pleural-pericardial effusion, atrial thrombus, and hepatomegaly; the animals that did not present signs of HF were excluded from the study. About 50% of the animals developed HF after surgery. At the end of 21 weeks, eighteen rats developed aortic stenosis (HFS group), which is a characteristic of HF. The HFS group was subdivided into two groups, as shown in Figure 1. The HFS/T4 group (9 rats) was administered T4 (L-T4 Sigma) at a physiological dose of 1.0 µg/100 g (16,17) of body weight (BW) subcutaneously, twice a day (7 am and 7 pm) for 5 days. This period is sufficient for the pharmacokinetics and pharmacodynamics of thyroxine (18,19). The HFS/S group (9 rats) was administered saline solution injections (0.9% NaCl, without T4) instead of T4. At the end of the treatment, all the animals were sacrificed.

Sham (n = 9), heart failure syndrome (HFS, n = 18), HFS/S administered saline 0.9% NaCl without T4 (n = 9), and HFS/T4 administered T4 at 1.0 µg/100 g BW (HFS/T4, n = 9).

### *Serca2* and *Ryr2* gene expression

Total RNA was extracted from cardiac tissue (left ventricle) using Trizol Reagent (Invitrogen), according to the manufacturer’s instructions. Complementary DNA (cDNA) was synthesized from 1000 ng of total RNA using the High Capacity cDNA Reverse Transcription Kit (Applied Biosystems, CA, USA). Real-time PCR (RT-qPCR) was used to quantitatively measure the messenger RNA (mRNA) levels of *Serca2a* (Rn00568762_m1) and *Ryr2* (Rn01470303_m1) using TaqMan Universal PCR Master Mix (Applied Biosystems, CA, USA) and the Applied Biosystems StepOne Plus detection system. Each sample was run in triplicate for RT-qPCR. Cycling conditions were as follows: enzyme activation at 50°C for 2 min; denaturation at 95°C for 10 min; cDNA amplification for 40 cycles of denaturation at 95°C for 15 s and annealing/extension at 60°C for 1 min. Cyclophilin (Rn00690933_m1) was used as the internal control and gene expression was quantified in relation to the values for the control groups (C15, C30, and C45, respectively) with the 2^-^ method (20).

### Serum analysis

Blood was collected in dry tubes and then centrifuged at 1008 g (Eppendorf Centrifuge 5804R) for 10 min for separating the serum from whole blood. Serum concentrations of free T3, free T4, and TSH were measured by Genese Laboratory-Botucatu-SP with the Luminex Corporation’s xMAP™ Tecnology, by immunofluorescence reaction.

### Statistical analysis

For biometric statistical analysis, the Student’s t-test (21) was used. Gene expression and hormone data were analyzed using analysis of variance (ANOVA) complemented by Bonferroni’s test. The data are expressed as mean ± standard deviation and a signiﬁcance level of 5% was adopted.  results

### Biometric and tissue wet/dry weight ratio data

The animals subjected to aortic stenosis surgery developed HF, established by the following findings: tachypnea, ascites, pleural effusion, atrial thrombus, and liver congestion (22-25). None of the nine sham group exhibited any of these clinical or pathological features. Biometric and tissue wet/dry weight ratio data for HFS and sham group are presented in Table 1. BW was greater in the sham group as compared to the HFS group. Normalized LV and RV (right ventricle) weights were greater in the HFS group than in the sham group. Lung wet/dry ratio was greater in the sham than the HFS group. No significant difference in liver wet/dry ratio was observed among the groups. The comparison of the groups HFS/S and HFS/T4 not showed significant statistical difference (data not shown) for biometric and tissue wet/dry weight ratio data.

**Table 1:** General characteristics of groups: Sham and HFS

	Sham (n = 9)	HFS (n = 18)
BW	452.62 ± 18.85^b^	348.89 ± 34.98^a^
LVW/BW	1.78 ± 0.13^a^	3.10 ± 0.30^b^
RVW/BW	0.51 ± 0.06^a^	0.95 ± 0.18^b^
Liver W/Dg/g	3.46 ± 0.79^a^	3.07 ± 0.58^a^
Lung W/Dg/g	4.54 ± 0.07^a^	5.24 ± 0.40^b^

Values are mean ± standard deviation n: number of rats; Sham: thoracotomy without the insertion of clip; HFS: rats with the insertion of a clip (aortic stenosis); BW: body weight; LVW: left ventricle weight; RVW: right ventricle weight. The averages followed by different alphabets (p < 0.01) show significance according to the Student’s t-test.

### Reduction in T3 levels after HF

The levels of thyroid hormones in the serum of the animals are presented in Table 2. Statistically significant reduction in the levels of T3, and no change in T4 and TSH levels was observed in the HFS/S group in comparison to the sham group, but exogenous administration of T4 led to a significant increase in T3 levels in the HFS/T4 group to the levels of the sham group.

**Table 2:** Hormonal measurement of free T3, T4, and TSH

Variable	Groups		

	Sham	HFS/S	HFS/T4
Free T3 (ug/mL)	12.13 ± 1.09^b^	9.44 ± 1.44^a^	12.05 ± 1.40^b^
Free T4 (ug/mL)	646.02 ± 27.53^a^	671.3 ± 49.25^a^	652.0 ± 131.50^a^
TSH (ug/mL)	5.16 ± 2.34^a^	4.98 ± 2.36^a^	5.68 ± 2.17^a^

TSH: thyroid stimulating hormone; T3: triiodothyronine; T4: thyroxine; Sham = thoracotomy without the insertion of a clip, HFS/S = aortic stenosis without T4 (saline, 0.9% NaCl), HFS/T4 = aortic stenosis with 1.0 µg T4/100 g BW. Data expressed as mean ± standard deviation. ANOVA complemented by Bonferroni’s test was used. Use of same alphabets represents p > 0.05; different alphabets represent p < 0.05.

### HF decreased *Serca2* and *Ryr2* levels, but T4 administration elevated them

**Figure 2:**
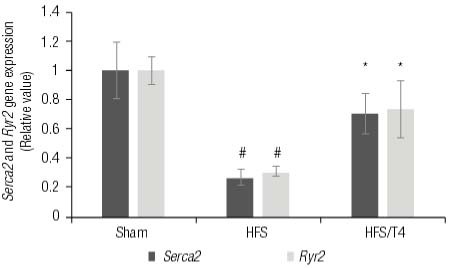
Gene expression of Serca2 and Ryr2 decreases in HF, while an increase is seen upon administration of T4.

RT-qPCR data provided a quantitative assessment of *Serca2* and *Ryr2* gene expression, illustrated in Figure 2. *Serca2* and *Ryr2* expression was reduced in the HFS group as compared to the sham group, while an increase in *Serca2* and *Ryr2* expression was observed in HFS/T4 group as compared to the HFS/S group.

Sham = thoracotomy without the insertion of a clip, HFS = aortic stenosis without T4, HFS/T4 = aortic stenosis with 1.0 µg T4/100 g BW. Data expressed as mean ± standard deviation. ANOVA was used, complemented by Bonferroni’s test. The # symbol represents the Sham group vs HFS (p < 0.001); the * symbol represents the HFS group vs HFS/T4 (p < 0.001).

## DISCUSSION

The role of thyroid hormone therapy is unclear in patients with HF and low serum T3 levels; this therapy, however, results in improvement in ventricular function. Some studies suggest the use of thyroid hormone analogs such as diiodothyropropionic acid and gene therapy along with T3 and/or T4 hormone replacement to modify thyroid hormone receptor or deiodinase expression and activity (26).

Using an experimental model as a base for studying ESS in HF, we were able to successfully induce ESS in animals with HF and at the same time demonstrate a reduction in the serum level of T3 in these animals. The decrease in T3 observed in our model is probably due D1, involved in the production of T3, as demonstrated by previous studies from our group. This study analyzed the conversion of T4 to T3 or rT3 in the liver and kidneys and found that animals with HF had a higher concentration of rT3 while no T3 was detected (27). The other possibility is the increase of D3 activity in the cardiomyocyties (28).

Our group has previously demonstrated that clipping surgery of the ascending thoracic aorta is an effective technique to induce HF and ESS in rats (27). These results are similar to those of Pimentel and cols*. *(29) and Hamilton (7), which demonstrated a correlation between the degree of HF and ESS; hence, the presence of ESS in HF patients is a prognostic factor in HF.

In the presence of ESS (reduced T3 levels), the gene expression of the intracellular calcium pumps is reduced. This results in altered intracellular calcium movement, which affects the contraction and excitation of the heart muscles, thus compromising the function of the heart (9,10).

Therefore, animals from the HFS group presented with ESS and showed decreasing *Serca2* and *Ryr2* gene expression. We showed the effect of treatment of HF with T4 (HFS/T4), in a physiological dose and verified the subsequent increase in expression of *Serca2* and *Ryr2* in the HFS/T4 group (Figure 1). This could be a result of conversion of administered T4 into T3; T3 is known to be essential for gene transcription of these pumps (9,10).

Some studies have suggested administration of T4 as a treatment for ESS that develops in acute kidney injury, in newborns with lung disease, and with the use of supraphysiological doses (12,13). The current study is significant primarily since we investigated the role of T4 in the treatment of ESS in HF, which is an understudied topic.

Treatment with T4 normalized T3 levels in the HFS/T4 group (Table 2) compared to those seen for HFS/S, thereby demonstrating that treatment with T4 can potentially reverse hormonal changes caused by ESS in HF. Previously four randomized controlled trials (RCTs) were conducted using either T4 or T3 for treatment of ESS. Two of these RCTs used T3 and the other two used T4 as the hormone for treatment of ESS. Three out of the four studies used these hormones in pharmacologic doses. All four studies showed that hormonal treatment further suppressed plasma TSH, and none of trials showed a therapeutic benefit to patients (30). In contrast, the present study demonstrated that T4 administration at a physiologic dose after HF normalizes T3 levels (HFS/T4) and does not suppress TSH secretion (Table 2). The normalization of serum T3 in HF rats, following T4 administration, resulted in a signiﬁcant increase in *Serca2* and *Ryr2* mRNA expression, thereby demonstrating the utility of this hormone to normalize molecular and hormonal changes produced by HF-induced ESS.

In conclusion, the T4 treatment can potentially normalize the levels of T3 as well elevated *Serca2* and *Ryr2* gene expression in the myocardium in heart failure rats with euthyroid sick syndrome.
